# Vermont State Medical Society

**Published:** 1867-06

**Authors:** 


Vermont State Medical Society.—The Medical Society of the State of Vermont met at Burlington on Wednesday, the 19th instant, and held its session for two days.

